# Mathematical artifacts in slope–intercept correlations invalidate biological inferences in aging research

**DOI:** 10.1073/pnas.2523907122

**Published:** 2025-12-08

**Authors:** Jacob A. Moorad

**Affiliations:** ^a^Institute of Ecology and Evolution, School of Biological Sciences, University of Edinburgh, Edinburgh EH9 3FL, United Kingdom

Cayuela et al. (1) interpret different aging trajectories between resident and migrating flamingos as support for trade-offs between early and late-life mortality using the Strehler-Mildvan correlation (SMC) (2), the mathematical association between intercept and the slope of a Gompertz model of aging. However, this correlation may not reliably indicate the underlying biology. Several studies assert that the SMC correlation can be artifactual (3, 4), with Burger and Missov (5) emphasizing that it “should not be used to diagnose any biological, physiological, or evolutionary processes.”

The fundamental concern is that slope–intercept correlations are mathematically constrained independent of biological trade-offs. While Blomqvist addresses measurement error in slope–intercept correlations (6), a statistical issue, this is not the problem identified here. The larger concern applies even to sophisticated analyses using mixed models like those of Cayuela et al.: this problem has no statistical solution. Even when population parameters are perfectly known, the slope–intercept correlation differs systematically from the early-late correlation (Table 1, General Formula) because aging rates depend inherently upon starting values.

**Table 1.** Mathematical foundation of slope-intercept correlation artifacts in aging research

Mathematical framework	Key insight
General formula $\frac{\rho_{12}\sigma_{2}-\sigma_{1}}{\sqrt{\sigma_{2}^{2}+\sigma_{1}^{2}-2\rho_{12}\sigma_{1}\sigma_{2}}}$	Slope-intercept correlations ρ_(z2 − z1), z1_ can differ systematically from early-late trait correlations ρ_12_. Trade-offs indicated only when ρ_12_ < 0.
Scenario 1: No cross-age correlations (ρ_12_ = 0)$\frac{-\sigma_{1}}{\sqrt{\sigma_{2}^{2}+\sigma_{1}^{2}}}$	Different variance structures generate different negative correlations absent any trade-offs. If σ_1_ > σ_2_, correlations approach −1; if σ_1_ < σ_2_, correlations approach 0.
Scenario 2: Equal variances (σ_1_ = σ_2_)$\frac{\rho_{12}-1}{\sqrt{2-2\rho_{12}}}$	Positive cross-age correlations still produce negative slope-intercept correlations. When ρ_12_ = 0: correlation = −0.71. As ρ_12_ increases toward +1, negative correlations weaken toward zero.
Cross-trait corollary $\frac{\rho_{a_{1},b_{2}}\sigma_{b_{2}}-\rho_{a_{1},b_{1}}\sigma_{b_{1}}}{\sqrt{\sigma_{b_{2}}^{2}+\sigma_{b_{1}}^{2}-2\rho_{b_{1},b_{2}}\sigma_{b_{1}}\sigma_{b_{2}}}}$	Mathematical artifacts persist when examining different traits. Aging as a derived measure conflates cross-trait correlations at late age (ρ_a1,b2_) with slope-intercept correlations.

Variables: z_1_, z_2_ = trait values at ages 1 and 2; σ_1_, σ_2_ = SD at ages 1 and 2; ρ_12_ = correlation between trait values at the two ages; a_1_ = trait A at age 1; b_1_, b_2_ = trait B at ages 1 and 2; ρ__a_1__,__b__2_ = cross-trait correlation between trait A at age 1 and trait B at age 2; ρ__a_1__,__b__1_ = cross-trait correlation at age 1; ρ__b_1__,__b__2_ = within-trait temporal correlation for trait B.

The issue is not statistical but conceptual: SMCs measure mathematical relationships between derived quantities, not biological trade-offs. While Cayuela et al. acknowledge that the SMC can be artifactual and call for cautious interpretation, their reasoning attaches biological significance to among-group variation in that correlation. Their perspective that SMCs should remain consistent across groups if merely artifactual overlooks how these correlations originate. I show that such results can be re-interpreted in two ways that do not involve trade-offs. Table 1 illustrates how variation in SMCs across groups can manifest without trade-offs when groups exhibit different patterns of heteroskedasticity across ages (early- vs. late-age trait variation; Scenario 1) or different degrees of positive correlations (anti-trade-offs; Scenario 2) between early and late traits.

This concern extends beyond the SMC to slope–intercept relationships involving aging. Aging is a derived measure (a difference); this complicates the interpretation of its variances and covariances (e.g., ref. 7). Formal evolutionary theory, including Williams’ antagonistic pleiotropy model of aging (8), views correlations between the same traits expressed early versus late in life as manifestations of trade-offs. However, studies commonly examine slope–intercept correlations, incorrectly interpreting negative associations as evidence for antagonistic pleiotropy (9). Comparing across different traits does not avoid the problem (see Table 1 Cross-trait Corollary). Interestingly, one of Williams’ more influential predictions was that “Rapid individual development should be correlated with rapid senescence.” (8, 10). While the biological mechanisms that underlie this prediction may exist, empirical tests of such predictions may present difficulties arising from the mathematical entanglements following from the slope–intercept correlations discussed above.

Treating aging as a fundamental rather than a derived trait leads to confusion between mathematical artifacts and evolutionary processes, a confusion that persists regardless of statistical sophistication, as demonstrated by Cayuela et al.’s analysis. The solution is to adopt direct tests of cross-age trade-offs (early-late age correlations rather than slope–intercept correlations) (e.g., ref. 9) to distinguish evolutionary mechanisms from mathematical artifacts.
